# Immunosurveillance by human γδ T lymphocytes: the emerging role of butyrophilins

**DOI:** 10.12688/f1000research.11057.1

**Published:** 2017-06-05

**Authors:** Dieter Kabelitz, Marcus Lettau, Ottmar Janssen

**Affiliations:** 1 Institute of Immunology, University of Kiel and University Hospital Schleswig-Holstein Campus Kiel, Arnold-Heller-Str. 3, Building 17, D-24105 Kiel, Germany

**Keywords:** lymphocytes, butyrophilins, Immunosurveillance, T-cell, immunotherapeutic

## Abstract

In contrast to conventional T lymphocytes, which carry an αβ T-cell receptor and recognize antigens as peptides presented by major histocompatibility complex class I or class II molecules, human γδ T cells recognize different metabolites such as non-peptidic pyrophosphate molecules that are secreted by microbes or overproduced by tumor cells. Hence, γδ T cells play a role in immunosurveillance of infection and cellular transformation. Until recently, it has been unknown how the γδ T-cell receptor senses such pyrophosphates in the absence of known antigen-presenting molecules. Recent studies from several groups have identified a unique role of butyrophilin (BTN) protein family members in this process, notably of BTN3A1. BTNs are a large family of transmembrane proteins with diverse functions in lipid secretion and innate and adaptive immunity. Here we discuss current models of how BTN molecules regulate γδ T-cell activation. We also address the implications of these recent findings on the design of novel immunotherapeutic strategies based on the activation of γδ T cells.

## Introduction

T lymphocytes are the specific effector cells of the adaptive immune system. T-cell differentiation takes place in the thymus, giving rise to large numbers of mature CD4 and CD8 T cells expressing a clonally variable αβ T-cell receptor (TCR). Interaction with thymic stromal cells, transcription factors, and cytokines together drive the differentiation of early thymic progenitor cells into mature CD4 and CD8 αβ T cells which recognize peptides presented in the context of major histocompatibility complex (MHC) class I (CD8 T cells) or class II (CD4 T cells) ^1^. The functional diversification of mature T cells into T helper type 1 (Th1), Th2, and Th17 cells and memory cell subsets is induced by the context-dependent interaction with neighboring cells (e.g. dendritic and epithelial cells) and transcriptional networks which are further modulated by metabolic and epigenetic processes ^2– 5^. In addition to these “conventional” T cells, T cells with a highly restricted canonical αβ TCR repertoire exist which recognize non-peptide antigens in the absence of restriction by classical MHC molecules. Such “unconventional” human αβ T cells include the invariant natural killer T (iNKT) cells expressing a Vα24-Jα18-encoded TCR and the mucosa-associated invariant T (MAIT) cells expressing a Vα7.2-Jα33-encoded TCR. iNKT cells recognize endogenous and exogenous (microbial) lipids presented by CD1 (specifically CD1d) molecules, whereas MAIT cells recognize small intermediates generated in the riboflavin (vitamin B2) metabolic pathway which are presented by the MHC-related 1 (MR1) molecules ^6^. Since unconventional αβ T cells are not dependent on the antigen processing machinery like conventional CD4 and CD8 T cells, they can rapidly perform effector functions upon ligand recognition. Both iNKT and MAIT cells are found in the blood and at increased numbers not only in mucosal tissue but also in the liver and are important players in local immunosurveillance and anti-bacterial immunity ^7, 8^. More recently, innate lymphoid cells (ILCs) have been identified as innate homologs of differentiated effector T cells which do not express clonally rearranged TCR but share similar transcription factor and cytokine specifications ^9^. Subsets of ILCs interact with innate and adaptive immune cells, epithelial cells, and microbiota and thereby contribute to tissue repair, metabolic homeostasis, and local inflammation ^10^.

While it might appear that the above outlined arsenal of available immune cells should suffice to combat all dangerous (infectious and non-infectious) antigens, evolution has conserved yet another class of unconventional T cells, i.e. T lymphocytes carrying a CD3-associated γδ TCR heterodimer rather than the αβ TCR. It has been known for a long time that the major population of γδ T cells found in the peripheral blood of adults specifically recognizes non-peptidic small microbial pyrophosphate molecules, again without requirement for a dedicated MHC class I, MHC class II, or CD1 presenting molecule ^11– 13^. It thus remained a mystery for many years how such “phosphoantigens” (pAg) contained in crude bacterial lysates ^14^, purified by preparative anion exchange chromatography ^15^, or chemically synthesized ^16^ can trigger such potent γδ T-cell responses.

A landmark paper addressing the activation requirements of human γδ T cells in response to pAg was published in 2012 by Harly and co-workers ^17^. These authors reported the unexpected finding that a member of the transmembrane butyrophilin (BTN) proteins was absolutely required for the activation of human γδ T cells by microbial or endogenous pAg. On the grounds of these findings, several groups set out to study the precise role of BTN proteins at the molecular level. Surprisingly, these investigations resulted in quite controversial models, assigning an essential role to either the extracellular ^18^ or the intracellular domain ^19^ of a particular BTN3A isoform. In this review, we discuss the current knowledge on the interplay of human γδ TCR with specific BTN proteins, both in terms of basic mechanisms of γδ T-cell activation and with respect to improving future strategies of γδ T-cell-based immunotherapies.

## γδ T cells: unconventional T lymphocytes linking innate and adaptive immunity

γδ T cells account for approximately 2–5% of peripheral blood T cells in healthy adult donors but are present at much higher numbers in mucosal tissues, where they comprise 20–30% of intraepithelial lymphocytes in the small intestine ^20^. In contrast to αβ T cells, there are only few variable (V) gene segments available in the germline genome which can be used during intrathymic TCR gene rearrangement to express functional TCR proteins. In humans, there are six expressed Vγ genes (Vγ2, 3, 4, 5, 8, and 9) and a similarly limited number of Vδ genes ^21^. Nonetheless, γδ TCR can display an enormous CDR3 loop diversity ^22^. γδ T cells expressing particular VγVδ pairing are not randomly distributed but are preferentially located in certain compartments. Thus, the majority of γδ T cells in peripheral blood express Vδ2 (paired almost exclusively with Vγ9), while intraepithelial γδ T cells frequently express Vδ1 (or other non-Vδ2 segments) which can pair with different Vγ elements ^13^. Most γδ T cells lack CD4 and CD8 surface expression, well in line with their MHC-independent ligand recognition (note, however, that a substantial proportion of γδ T cells can express CD8 at low levels). Major efforts were made over the years to identify antigens and ligands that are specifically recognized by the γδ TCR ^22, 23^. A list of some currently identified ligands for human γδ T cells is presented in Table 1. Obviously, the best-characterized ligands are prokaryotic and eukaryotic pAg, which are exclusively recognized by human Vδ2Vγ9 γδ T cells ^24^. Such pyrophosphates are intermediates of the eukaryotic mevalonate or the prokarytic non-mevalonate (also termed Rohmer’s) pathway of isoprenoid synthesis ^15, 25, 26^. Other ligands for Vδ2Vγ9 T cells include the ectopically expressed DNA mismatch repair protein hMSH2 ^27^ and F1-ATPase together with apolipoprotein A-I ^28^. Some of the identified ligands for non-Vδ2 γδ TCR include endothelial protein C receptor ^29^ and lipids bound to CD1d ^30^ but also the stress-inducible MHC class I-related chain A (MICA) molecules ^31^. In all instances, recognition of respective ligands by the γδ TCR is a rapid event and addresses all γδ T cells carrying the appropriate TCR with little, if any, contribution of CDR3 variation. Since Vδ2Vγ9 cells comprise the vast majority (up to 95%) of peripheral blood γδ T cells and all respond to pAg stimulation with no need for antigen processing, this implies that a large proportion (2–4%) of all peripheral blood T cells is rapidly activated (e.g. to produce cytokines including interferon-γ and tumor necrosis factor-α) upon encounter of such pAg ^13^.

**Table 1.** Some examples of ligands that are specifically recognized by subsets of human γδ T cells.

γδTCR	Ligand	References
Vδ2Vγ9	Prokaryotic pAg, HMBPP Eukaryotic pAg, IPP F1-ATPase + apolipoprotein A-I hMSH2	15 26 28 27
Vδ1	MICA CD1d-lipid	31 30
Vδ5	EPCR	29

Abbreviations: EPCR, endothelial protein C receptor; HMBPP, (E)-4-hydroxy-3-methyl-but-2-enyl pyrophosphate; hMSH2, human MutS homolog 2; IPP, isopentenyl pyrophosphate; MICA, major histocompatibility complex class I-related chain A; pAg, phosphoantigen

In addition to the TCR, γδ T cells express other activating cell surface receptors, notably natural killer group 2 member D (NKG2D), which is a receptor for multiple stress-inducible MHC class I-related molecules including MICA/B and six members of the UL16 binding protein family (ULBP1-6) ^32^. NKG2D is expressed on innate natural killer (NK) cells, some CD8 and CD4 T cells, and essentially all γδ T cells. While normal cells usually do not express NKG2D ligands, cell surface expression is induced by cell stress, DNA damage, and cellular transformation. Upon ligand binding, NKG2D transmits cellular activation via the PI3-kinase pathway, resulting in cytokine production and triggering of cytotoxic activity ^33^. NKG2D ligands can be released from the surface of tumor cells via protease-mediated shedding or via exosome secretion, and soluble NKG2D ligands may block NKG2D receptor activation and thereby serve as a tumor immune escape mechanism ^34^.

**Figure 1.**
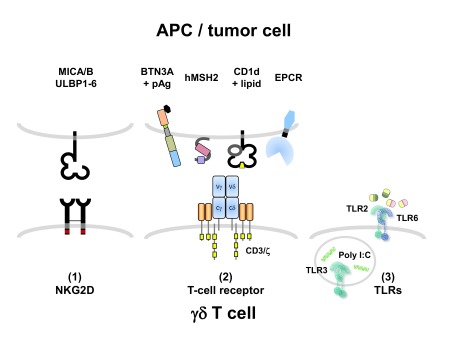
Three classes of receptors regulating human γδ T-cell activation. (1) The activating natural killer (NK) receptor NK group 2 member D (NKG2D) recognizes stress-inducible ligands including major histocompatibility complex class I-related chain A (MICA)/MICB and UL16 binding protein family (ULBP) 1–6 and triggers cytotoxic activity and cytokine production via the phosphoinositide 3 (PI3)-kinase pathway. (2) The CD3-associated T-cell receptor (TCR) recognizes ligands including “phosphoantigens” (pAg) in a butyrophilin (BTN) 3A-dependent way and human MutS homolog 2 (hMSH2) (Vδ2Vγ9 TCR), or lipids bound to CD1d and endothelial protein C receptor (EPCR) (non-Vδ2 TCR). (3) Pattern recognition receptors including Toll-like receptors (TLRs) sense conserved microbial ligands such as acetylated lipids (TLR2/6 heterodimer) or poly I:C (intracellular TLR3) and co-stimulate γδ T-cell activation via the nuclear factor kappa-light-chain-enhancer of activated B cells (NF-κB) pathway. APC, antigen-presenting cell.

Furthermore, γδ T cells can also express some receptors specifically associated with the innate immune system, notably Toll-like receptors (TLRs), and corresponding TLR ligands can co-stimulate γδ T-cell activation ^35^. Conceivably, such effects might be primarily mediated via monocytic and/or dendritic cells when heterogeneous cell populations are investigated ^36^, but it has also been demonstrated that purified γδ T cells express certain TLRs and directly respond to TLR ligand co-stimulation ^37, 38^. To summarize, γδ T cells express receptors of both the innate (e.g. NKG2D and TLR) and the adaptive (TCR) immune system, and the outcome of functional responses is regulated through integration of various signaling pathways ( Figure 1). There is another feature of human Vδ2Vγ9 T cells which further places them as a link between the innate and adaptive immune systems: as initially reported by Bernhard Moser’s group, activated γδ T cells can serve as antigen-presenting cells to specifically stimulate peptide-specific αβ T cells ^39^. Importantly, γδ T cells can even take up antigen particles, process such antigens intracellularly, and load corresponding peptides onto MHC class I molecules for cross-presentation to antigen-specific CD8 αβ T cells, a process normally restricted to “professional” antigen-presenting cells such as dendritic cells ^40^. The antigen-presenting capacity of γδ T cells may help to initiate a subsequent tumor antigen-specific CD8 T-cell response once γδ T cells have killed opsonized tumor cells and taken up apoptotic tumor cell fragments ^41^.

## γδ T cells: important players in anti-tumor immunity

Many solid tumors and leukemia/lymphoma cells are quite susceptible to γδ T-cell-mediated lysis. In contrast to αβ T cells, γδ T cells recognize tumor cells not on the basis of tumor-specific antigenic peptides presented in the context of MHC class I or class II molecules but rather sense cell-surface-expressed stress molecules and/or metabolites of the dysregulated mevalonate pathway. Moreover, γδ T cells can make use of both the TCR and the NKG2D receptor to recognize and kill tumor cells ^42, 43^. Interestingly, the sensitivity of tumor cells to lysis by Vδ2Vγ9 γδ T cells can be pharmacologically manipulated. Nitrogen-containing bisphosphonates (N-BPs) such as zoledronic acid are in clinical use to treat diseases associated with bone resorption. In addition to their anti-resorptive bone activity, N-BPs also interfere with the mevalonate metabolic pathway where γδ T-cell-stimulating pyrophosphates are generated ^25^. N-BPs block an enzyme downstream of the synthesis of isopentenyl pyrophosphate (IPP), leading to increased accumulation of IPP and thereby to γδ T-cell activation ^44^. Therefore, pretreatment of tumor cells with N-BP increases their susceptibility to γδ T-cell-mediated lysis ^45^. Application of N-BPs to patients also induces *in vivo* activation of γδ T cells ^46^, and in fact some clinical responses have been noted in small-scale studies in cancer patients given intravenous N-BPs together with low-dose interleukin-2 ^47^. Moreover, γδ T cells have also been adoptively transferred to cancer patients, with no obvious major adverse effects but some clinical responses in a few patients ^47^. The efficacy of tumor cell killing by γδ T cells can be further increased by specifically targeting γδ T cells to tumor cells via antibody-mediated cellular cytotoxicity (ADCC) ^48^ or bispecific antibody constructs ^49, 50^. While Vδ2Vγ9 γδ T cells can be easily activated and expanded to large cell numbers by activation with pAg or N-BPs, it should be kept in mind that non-Vδ2 subsets of γδ T cells might also have potent anti-tumor activity, and protocols for selective expansion of those γδ T cells are in development ^51^. Attempts to explore the anti-tumor capacity of γδ T cells in a clinical setting were boosted by the recent demonstration in a large patient cohort that the proportion of γδ T cells among tumor-infiltrating immune cells was the best positive predictive parameter across a multitude of human tumor entities ^52^. On the other hand, however, it must be considered that γδ T cells might also negatively regulate anti-tumor immune responses. For instance, it has been demonstrated that γδ T cells infiltrating into human breast cancer have a regulatory activity and inhibit αβ T-cell responses ^53^. Moreover, other potentially tumor-promoting activities of γδ T cells have been reported in colorectal and pancreatic cancer ^54, 55^. Overall, however, it appears that γδ T cells are interesting and promising candidates for cellular immunotherapy supplementing other strategies such as NK cells and chimeric antigen receptor (CAR) T cells ^56, 57^.

## Butyrophilins: a large family of proteins with immunomodulatory functions

**Figure 2.**
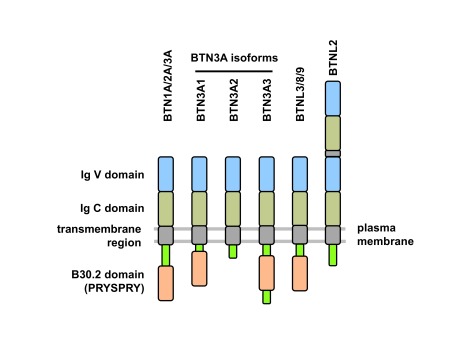
Domain structure of butyrophilin (BTN) proteins. Typically, BTN and BTN-like (BTNL) proteins consist of extracellular immunoglobulin V (IgV)- and IgC-like domains, a transmembrane domain, and a cytosolic B30.2 (or PRYSPRY) domain. Three isoforms of BTN3A differ in their cytosolic structure (BTN3A1: B30.2 domain; BTN3A2: no B30.2 domain; BTN3A3: B30.2 domain plus additional stretch of amino acids). BTNL2 has two tandem repeats of IgV and IgC domains and lacks the cytosolic B30.2 domain.

BTNs were originally described as plasma-membrane-associated glycoproteins in the lactating mammary glands of many species which constitute a major component of the milk fat globule membrane ^58^. The type 1 transmembrane BTN proteins belong to the immunoglobulin (Ig) superfamily and typically consist of extracellular Ig-like domains (IgV and IgC), a transmembrane domain, and, in some but not all cases, an intracellular B30.2 signaling domain ^59– 61^. BTN and BTN-like (BTNL) proteins are variably related to the B7 family of costimulatory molecules (e.g., CD80 and CD86) which supports the role of (at least) some BTN members in the immune system ^62^. The genes are clustered in two regions on human chromosome 6: *BTN* telomeric to HLA class I genes and *BTNL* near the HLA-DR genes. An additional *BTNL* gene cluster is located on human chromosome 5q35 ^61^. The protein domain structure of some functionally important BTN and BTNL members is shown in Figure 2. The cytosolic B30.2 domain (also termed PRYSPRY) and the homologous SPRY domain are present in many cellular proteins, including tripartite motif molecules (TRIM), where they potentially interact with diverse intracellular molecules including NOD2, retroviral capsids, or Fc parts of IgG ^63, 64^. Given that BTN molecules have multiple roles in innate and adaptive immunity, it comes as no surprise that *BTN* gene polymorphisms may influence disease susceptibility. As an example, *BTN3A2* has been shown to be associated with susceptibility to type I diabetes ^65^, and more examples are discussed in 60. Interestingly, genetic variants in *BTN* genes can also alter susceptibility to infection, as has been demonstrated for a selection of hepatitis C virus genotypes and subsequent disease progression ^66^.

BTN proteins are widely expressed in immune cells and epithelial cells and can exert a multitude of immunoregulatory activities ^60, 61^. While a few binding partners have been identified (such as DC-SIGN [CD209] on dendritic cells and monocytes for BTN2A1 ^67^), specific receptors are largely unknown. It also appears that the overall effect of specific BTN molecules depends on experimental conditions and respective reagents (recombinant proteins, soluble or immobilized antibodies, cell-surface-expressed molecules, etc.). The extracellular domain of the human BTNL protein BTNL8 co-stimulates proliferation and cytokine production of anti-CD3 antibody-stimulated CD4 and CD8 αβ T cells, and a putative BTNL8 receptor on the cell surface of resting T cells was detected by flow cytometry with a BTNL8-Fc fusion protein ^68^. Olive’s group has generated a number of monoclonal antibodies (mAb) directed against the extracellular domain of BTN3A molecules (also termed CD277), which do not differentiate between the three isoforms BTN3A1, BTN3A2, and BTN3A3 ^62^. They showed that anti-CD277 mAb clone 20.1 co-stimulated cytokine production and early signaling cascades in purified human CD4 and CD8 T cells when immobilized together with anti-CD3 mAb in cell culture plates or on microbeads, pointing to an αβ T cell co-stimulating activity of BTN3A ^69^. In contrast, another anti-CD277 mAb specific for a different epitope in the extracellular region of BTN3A (clone 232-5) rather inhibited T-cell activation when added to anti-CD3 mAb activated CD4 or CD8 T cells ^70^. While these studies indicated that CD277 expressed on T cells can transmit positive or negative co-stimulatory signals, other studies showed that CD277 overexpressed on antigen-presenting cells profoundly inhibited T-cell proliferation and cytokine production ^71^. Using a Myc-tagged extracellular CD277 construct, Cubillos-Ruiz also obtained evidence for the expression of a CD277-binding protein on activated but not resting human T cells ^71^. Together with their observation of strong CD277 expression on most of the analyzed ovarian cancer tissues, these authors argued that CD277 is a negative regulator of human T-cell activation with relevance for the immunosuppressive tumor micromilieu ^71^. This view, however, does not integrate the fact that T cells themselves (like most, if not all, other immune cells) strongly express CD277 ^62, 69, 72^.

Other BTN/BTNL members with reported modulatory activity on αβ T-cell activation include BTN2A2 and BTNL2. The extracellular part of BTN2A2 (i.e. a BTN2A2-Fc fusion protein) inhibited early signaling events in anti-CD3 plus anti-CD28 mAb stimulated murine T cells and prevented cell cycle entry. Interestingly, BTN2A2-Fc also induced *de novo* expression of FoxP3 in anti-CD3/anti-CD28 mAb activated naive CD4 T cells, suggesting that BTN2A2 may attenuate T-cell activation through multiple pathways, including the induction of FoxP3-expressing regulatory T cells (Tregs) ^73^. Recently, *BTN2A2* ^–/–^ mice were reported to exhibit enhanced T-cell responses as shown by greater severity of T-cell-dependent models of autoimmunity (experimental autoimmune encephalomyelitis [EAE]) but also enhanced response to tumor vaccination ^74^. Quite similar to BTN2A2, BTNL2 also inhibits murine T-cell activation and co-stimulates FoxP3 induction and thus Treg induction when applied as immobilized BTNL2-Fc fusion protein ^75^. Interestingly, *BTNL2* was found to be upregulated during acute-phase malaria infection, pointing to a possible feedback loop between inhibitory BTN/BTNL molecules and T-cell activation in inflammation and infection ^76^.

In addition to immune cells, BTN and BTNL proteins are expressed in the intestine and regulate tissue integrity, local immune responses, and inflammation. Recent gene expression data point to a correlation of upregulated intestinal *BTN*/ *BTNL* gene expression with inflammatory bowel diseases, in line with an important role of BTN/BTNL proteins in shaping local T-cell responses ^77^. In murine intestinal epithelial cells, BTNL1 and BTNL6 form heteromeric complexes which enhance the proliferative activity of intraepithelial lymphocytes, specifically of a subset of γδ T cells expressing the Vγ7Vδ4 TCR ^78, 79^. Interestingly, it was recently shown that human gut epithelial cells express BTNL3 and BTNL8, which together also regulate tissue-specific γδ T cells, in this case intestinal γδ T cells expressing a Vγ4 TCR ^80^. Taken together, it is obvious that BTN and BTNL proteins regulate multiple T-cell responses in a negative or positive manner. One of the unsolved questions here is how such signals are transmitted to T cells (i.e. the nature of putative receptors), an issue which needs further investigation.

## The puzzling role of BTN3A1 in γδ T-cell activation

**Figure 3.**
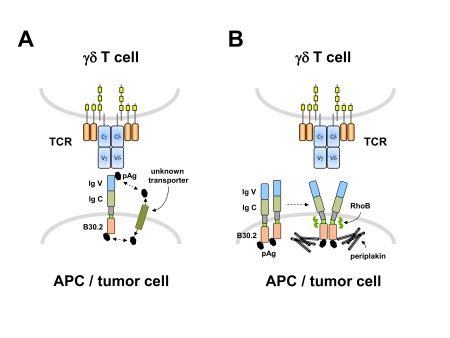
Alternative models of the role of butyrophilin (BTN) 3A molecules in phosphoantigen (pAg)-mediated γδ T-cell activation. **A**: pAg directly bind to the extracellular immunoglobulin V (IgV) domain of BTN3A1 and are then “presented” to the γδ TCR; as-yet-unidentified transporter molecules would shuffle pAg (generated within tumor cells due to the dysregulated mevalonate pathway) from the cytosol to the extracellular compartment for presentation by BTN3A1 ^18, 87^. **B**: pAg bind to the cytosolic B30.2 domain, leading to the recruitment of linker proteins including periplakin and the activation of the RhoB GTPase, which together induce a spatial redistribution of BTN3A1 molecules recognized by the γδ TCR ^19, 85, 91^. APC, antigen-presenting cell; TCR, T-cell receptor.

As already mentioned, an indispensable role of CD277/BTN3A in the activation of human Vδ2Vγ9 γδ T cells by microbial or tumor-derived pAg was reported by Harly and colleagues ^17^. These authors used CD277 knockdown and domain-shuffling strategies to demonstrate the importance of the BTN3A1 isoform (carrying the cytosolic B30.2 domain) in this process. They went on to show that the anti-CD277 mAb 20.1 (used in immobilized form in previous studies to demonstrate co-stimulatory activity on CD4 and CD8 αβ T cells ^69^) could selectively activate Vδ2Vγ9 T cells when added in soluble form together with interleukin-2 to peripheral blood mononuclear cells, and furthermore sensitized a broad range of tumor cells to killing by γδ T cells ^17^. They also identified another anti-CD277 antibody termed 103.2, which binds to a different epitope on the extracellular part of BTN3A and specifically inhibited Vδ2Vγ9 T-cell activation by pAg, N-BP, or agonistic mAb 20.1 ^17, 81^. The essential role of BTN3A1 for pAg stimulation of human γδ T cells was confirmed by several other reports ^18, 19, 82– 86^. While the original study by Harly *et al*. described the importance of BTN3A/CD277 for human γδ T-cell activation by pyrophosphate antigens, the molecular mechanism was not yet precisely identified. Subsequently, two largely conflicting models were proposed, i.e. a “presenting” versus a “pyrophosphate sensing” function of CD277 ( Figure 3). Vavassori and co-workers reported that pAg IPP and HMBPP could directly bind with low affinity to the recombinant extracellular IgV domain of BTN3A1; furthermore, they also observed weak binding of recombinant soluble Vδ2Vγ9 TCR to immobilized BTN3A1 molecules, which was further facilitated by IPP ^18^. These observations were in line with BTN3A1 serving as an antigen-presenting molecule for pAg to be specifically recognized by the human Vδ2Vγ9 TCR ( Figure 3A ^18, 87^). However, direct binding of pAg to the extracellular domain of BTN3A1 could not be verified by other groups ^19, 82, 84^. Instead, Sandstrom and co-workers demonstrated that the cytosolic B30.2 domain could directly bind several γδ T-cell-stimulating pAg through a positively charged surface pocket ^19^, an observation which was confirmed by others ^84– 86, 88^. Currently, most available data thus support the pyrophosphate-sensing function of the cytosolic B30.2 domain ^89, 90^. How, then, can binding of pyrophosphates to the cytosolic domain of a transmembrane protein (BTN3A1) translate into TCR-dependent selective activation of a specific (Vδ2Vγ9) γδ T-cell subset? Recent progress in the field has helped to elucidate some of the molecular mechanisms. Overall, it appears that an “inside-out” signaling mechanism induced by intracellular pAg sensing conveys a spatial redistribution or conformational change of the extracellular CD277 domain, which is then somehow recognized by the Vδ2Vγ9 TCR ^89, 91^. Recently, crucial steps along this pathway have been identified. The cytoskeletal adaptor protein periplakin has been shown to interact with a membrane-proximal intracellular part of BTN3A1 ^85^. Periplakin is a member of a family of cytoskeletal linker proteins that interact with various membrane-associated proteins and are involved in cytoskeletal (re)organization ^92^. It is thus conceivable that upon pAg binding the interaction of cytosolic parts with periplakin and possibly additional adaptor proteins contributes to the spatial rearrangement of BTN3A1 ^85^. Another step in this process is the small GTPase RhoB, which was recently identified in a genome-wide screen as an important component in BTN3A1-dependent tumor cell recognition by Vδ2Vγ9 T cells ^91^. RhoB interacts with and regulates the membrane mobility of BTN3A1, and intracellular redistribution of RhoB in different tumor cells correlated with their recognition by γδ T cells. These results point to a correlation in tumor cells between the dysregulated mevalonate pathway (which also controls small GTPases), RhoB activity, accumulation of pyrophosphates, and sensitivity to γδ T-cell killing ^91^.

Taken together, it is now clear that BTN3A/CD277 is required for pAg-mediated activation of Vδ2Vγ9 γδ T cells and that periplakin and RhoB have important roles in spatial rearrangement of BTN3A1 following intracellular pAg sensing ( Figure 3B). However, some pieces of the puzzle are still unsolved. While a membrane reorganization of BTN3A1 (which can apparently also be induced by agonistic anti-CD277 antibodies ^89^) is a crucial step, it is not yet known what precisely then the γδ TCR recognizes. Moreover, and in contrast to initial studies ^17^, it appears that it is not just the BTN3A1 isoform and its cytosolic B30.2 domain which are involved in pAg-mediated γδ T cell activation—BTN3A2 and BTN3A3 isoforms have been implicated ^85^. Furthermore, using Chinese hamster ovary cells retrovirally transduced with the human *BTN3A1* gene and additionally harboring, or not, human chromosome 6, Riaño and co-workers obtained evidence for a role for additional genes in the BTN3A1-dependent activation of γδ T cells by pAg ^83^. Therefore, we can expect to witness the discovery of additional new players before we fully understand how cell-surface-rearranged BTN3A molecules and pAg exclusively activate human Vδ2Vγ9 T cells. Along this line, it will be important to study in more detail the role of various accessory cells in this process. BTN3A is widely expressed on leukocytes, yet only monocytes serve as accessory cells for three mechanistically different stimuli for Vδ2Vγ9 T cells, i.e. N-BP, pAg, and agonistic anti-CD277 mAb 20.1 ^72^. In co-cultures with purified γδ T cells, purified CD277-positive CD4 T cells can “present” pAg HMBPP to γδ T cells ^72^. Given that HMBPP is most likely not directly presented by extracellular BTN3A1 domains ^89, 90^, how then do pAg enter the cell to initiate γδ T-cell activation following binding to cytosolic B30.2? A putative transporter molecule has been postulated ^87^ ( Figure 3A) and recently an energy-dependent uptake of HMBPP was demonstrated ^86^, but the precise molecular mechanisms remain to be clarified.

## Concluding remarks

BTN and BTNL have emerged as potent immunomodulatory proteins. The T-cell-inhibitory activity of some BTN/BTNL members suggests that they might be novel targets for checkpoint inhibitors, in addition to established checkpoint proteins such as CTLA-4, PD1, and PD-L1 ^93^. Some BTN/BTNL proteins have a unique role in recruiting and activating particular subsets of unconventional γδ T cells. The recently discovered role of BTNL3 and BTNL8 for shaping Vγ4 γδ T cells in the human gut might be relevant for the loss of mucosal barrier function in inflammatory bowel diseases. The perhaps most fascinating example is the *ménage à trois* of BTN3A, pyrophosphate molecules, and the human Vδ2Vγ9 TCR. The availability of anti-BTN3A/CD277 antibodies which selectively activate (e.g. mAb 20.1) or inhibit (e.g. mAb 103.2) Vδ2Vγ9 γδ T cells opens new avenues for γδ T-cell-directed immunotherapies. In a pre-clinical xenotransplantation model of acute myeloid leukemia, therapeutic application of mAb 20.1 enhanced the therapeutic efficacy of adoptively transferred Vδ2Vγ9 T cells ^94^. Therefore, humanized agonistic anti-BTN3A/CD277 antibodies might be a novel and highly specific approach to activate tumor-reactive γδ T cells *in vivo*. Vice versa, humanized inhibitory anti-BTN3A/CD277 antibodies might represent potent reagents for selective silencing of Vδ2Vγ9 T cells in clinical conditions where they might contribute to the disease process, e.g. in autoimmune diseases ^95^.
